# Ultrasonographic evaluation of the normal gastrointestinal wall in dogs and cats: a systematic review on study design and imaging outcomes

**DOI:** 10.1080/01652176.2026.2622732

**Published:** 2026-02-09

**Authors:** Marisa Esteves-Monteiro, Margarida Duarte-Araújo, Clara Landolt, Cláudia S. Baptista

**Affiliations:** a Associated Laboratory for Green Chemistry of the Network of Chemistry and Technology (LAQV@REQUIMTE), University of Porto (UP), Porto, Portugal; b Department of Immuno-Physiology and Pharmacology, School of Medicine and Biomedical Sciences (ICBAS), University of Porto (UP), Porto, Portugal; c Laboratory of Pharmacology, Department of Drug Sciences, Faculty of Pharmacy, University of Porto (FFUP), Porto, Portugal; d Veterinary Hospital of the University of Porto (UPVet), ICBAS-UP, Porto, Portugal; e Department of Veterinary Clinics, ICBAS-UP, Porto, Portugal; f AL4AnimalS, Center for the Study of Animal Science (CECA), University of Porto (UP), Porto, Portugal

**Keywords:** Canine, feline, diagnostic ultrasound, disease-free gastrointestinal wall, review

## Abstract

Diagnostic ultrasound (US) is a noninvasive, cost-effective imaging modality widely used for evaluating the gastrointestinal (GI) tract in companion animals. It provides information on wall thickness and layer differentiation, allowing assessment of normal anatomy and pathological changes. Despite its diagnostic relevance, ultrasonographic reference values for the GI tract in dogs and cats remain inconsistent across publications. This study reviewed ultrasonographic characteristics of the normal GI wall in dogs and cats and compiled a consensus-based reference table for overall wall thickness and individual layer proportions to enhance clinical interpretation. A literature search of PubMed and Scopus identified studies assessing the ultrasonographic features of normal GI segments, from stomach to colon, in healthy dogs and cats. Twelve studies met the inclusion criteria: six focused on dogs and six on cats. Reference values for GI wall thickness and its layers were reported in both species. However, discrepancies were noted in weight-based classifications for dogs, and the stomach of adult dogs remains poorly studied. Moreover, evaluation of gastric rugal and inter-rugal folds remains limited in this species. US is valuable for GI assessment, but dispersion of reference values across studies may hinder accessibility. Establishing standardized ultrasonographic parameters could improve diagnostic accuracy and clinical decision-making.

## 1. Introduction

Diagnostic ultrasound (US) is a noninvasive, cost-effective, readily accessible medical imaging technique that utilizes high-frequency sound waves to generate real-time images of the body (Morris and Perkins 2012). This versatile imaging modality plays a pivotal role in clinical practice, offering valuable diagnostic insights across a broad spectrum of medical applications (Meomartino et al. 2021). Among its many uses, US has proven to be an essential tool in the assessment, diagnosis, treatment, and ongoing management of various medical conditions, including those affecting the gastrointestinal (GI) tract (Larson and Biller 2009).

Within the GI system, US provides a wealth of information that is critical for evaluating both normal and pathological conditions. This includes assessing wall thickness and its individual layers, characterizing luminal contents, evaluating motility, and visualizing adjacent organs and structures such as the pancreas, mesentery, lymph nodes, and peritoneum (Morris and Perkins 2012; Seiler et al. 2022). A systematic approach to scanning the GI tract ensures a comprehensive evaluation, with key segments routinely examined including the stomach, pyloroduodenal junction, duodenum, jejunum, ileum, ileocecocolic junction, and colon (Seiler et al. 2022).

A thorough understanding of both normal and abnormal ultrasonographic presentations of the GI tract in companion animals, such as dogs and cats, significantly enhances diagnostic accuracy when investigating GI disorders (Larson and Biller 2009). The most common diseases or conditions that may affect the normal thickness and/or layering of the GI tract wall include inflammation, chronic enteropathies, neoplastic and non-neoplastic disorders, ulceration, and rupture (Manczur and V 2000). These alterations, commonly seen in both dogs and cats, often lead to changes in echogenicity and/or thickness of the intestinal wall and may selectively involve some intestinal layers (Diana et al. 2009; Zwingenberger et al. 2010; Laurenson et al. 2011; Page et al. 2021).

Given the diagnostic significance of intestinal wall thickness and its layered structure, it is imperative to establish robust and standardized reference values for the ultrasonographic appearance of the entire GI tract. This includes defining normal full-thickness measurements and quantitatively characterizing the proportions of each layer (Seiler et al. 2022). Standardized imaging protocols and validated reference values facilitate objective assessments, improve diagnostic consistency, and enhance the ability to detect and monitor disease progression. By refining these parameters, veterinary professionals can optimize the use of US in the evaluation of GI health and disease, ultimately leading to more accurate diagnoses and improved patient outcomes.

We hypothesized that miscellaneous material and methods may influence consensual and objective imaging outcomes. Accordingly, the aims of this study were to: 1) provide a contemporary systematic review of the qualitative and quantitative ultrasonographic features of the normal GI tract wall in dogs and cats; 2) develop a collective imaging feature-based table with consensus intervals for the overall wall thickness and corresponding individual layers of the GI tract in dogs and cats, to facilitate US features of the GI tract interpretation in clinical practice, and 3) to identify existing knowledge gaps in the US features of the normal GI tract in companion animals.

## 2. Material and methods

### 2.1. Literature search and search terms

The Pubmed and Scopus databases were searched from their inception until the most recent search on [17/02/2025], to identify all papers related to US of the GI tract in dogs and cats. The search terms used in the databases included: [ultrasound OR sonography OR ultrasonography (MeSH terms)] AND [gastrointestinal tract (Mesh terms) OR intestinal OR colon (MeSH terms) OR stomach (MeSH terms)] AND [dog OR cat (MeSH terms) OR canine OR feline OR pets (MeSH terms)].

### 2.2. Inclusion and exclusion criteria

According to the inclusion criteria, studies were eligible if: i) were published in English or Portuguese; ii) had full text availability; iii) were performed in clinically healthy canine and feline patients without any history of GI disease; iv) evaluated any segment of the GI tract, from stomach to colon, *via* abdominal US, v) reported quantitative measurements of GI wall thickness (either full thickness or individual layers), and vi) were original research articles. Regarding the exclusion criteria, studies were rejected if they were: i) reviews or systematic reviews; ii) performed in humans, laboratory animals or other animal species; iii) studies without quantitative ultrasonographic measurements, and iv) papers without full-text availability. Finally, to ensure that no relevant papers were unidentified, the reference lists of all the selected papers were scrutinized.

For each study, the criteria used by the authors to classify animals as ‘healthy’ were recorded (e.g. physical examination, hematology, biochemical analysis, fecal examination, histopathology when available). When the definition of health status was unclear or insufficiently described, this was considered a potential source of bias and is discussed as a limitation.

### 2.3. Study selection

All studies identified in the search were assessed by title and abstract by two authors working independently (MEM and CSB) and after the individual selection, disagreements were solved by consensus or through the final decision of a third and fourth author (MDA and CL). Those that were irrelevant based on title, abstract or study type (case reports, letters to the editor, comments, or review papers) were excluded in this phase of initial screening. The remaining papers were evaluated by their full text for their appropriateness to the inclusion criteria by the same two authors. Once again, in case of disagreement, a third author had the final decision (MDA).

### 2.4. Data review process and analysis of studies

For each study the following data was collected: i) study population (species, gender, age, weight, number of patients, assessment of normal status, alive/cadaver); ii) study design (type of study, animal awake/sedated/anesthetized, degree of stomach distension, full thickness/individual layering measurements, probe frequency, imaging planes, variables assessed, present/absent histopathologic evaluation); iii) imaging outcomes; iv) conclusions, and v) limitations of the study. Whenever available, 95% confidence intervals reported in the original studies were extracted and are presented in Tables 3–5. No additional inferential statistical analyses were conducted, as this review aimed to provide a narrative synthesis of published reference values. The results were compiled descriptively to offer a systematic and transparent overview of the available evidence.

**Table 3.** Range of normal gastric and intestinal segment full-wall and individual layers thickness, in adult dogs grouped by weight, according to the literature reviewed.

GI segment	Dog wall range (mm)		
	<15kg	15–30 kg	>30 kg
Stomach			
Full thickness	3.00 (trans.) and 3.30 (long.) (Penninck et al. 1989)	3.00 (trans.) and 3.25 (long.) (Penninck et al. 1989)	4.00 (trans. and long.) (Penninck et al. 1989)
Duodenum			
Full thickness	3.8 ± 0.5 [2.9–4.7] (Gladwin et al. 2014)	4.1 ± 0.7 [3.0–5.5] (Gladwin et al. 2014)	4.4 ± 0.7 [3.1–5.7] (Gladwin et al. 2014)
Layer thickness			
Mucosa	2.4 ± 0.5 [1.6–3.5] (Gladwin et al. 2014)	2.6 ± 0.6 [1.5–3.7] (Gladwin et al. 2014)	2.8 ± 0.5 [2.0–3.9] (Gladwin et al. 2014)
Submucosa	0.6 ± 0.1 [0.3–0.8] (Gladwin et al. 2014)	0.6 ± 0.2 [0.3–1.0] (Gladwin et al. 2014)	0.6 ± 0.2 [0.3–1.2] (Gladwin et al. 2014)
Muscularis	0.5 ± 0.1 [0.2–0.8] (Gladwin et al. 2014)	0.5 ± 0.1 [0.3–0.8] (Gladwin et al. 2014)	0.6 ± 0.2 [0.2–0.9] (Gladwin et al. 2014)
Serosa	0.4 ± 0.1 [0.2–0.6] (Gladwin et al. 2014)	0.4 ± 0.1 [0.3–0.6] (Gladwin et al. 2014)	0.4 ± 0.1 [0.2–0.7] (Gladwin et al. 2014)
Jejunum			
Full thickness	3.0 ± 0.5 [2.2–4.1] (Gladwin et al. 2014)	3.5 ± 0.5 [2.4–4.8] (Gladwin et al. 2014)	3.8 ± 0.4 [2.7–4.7] (Gladwin et al. 2014)
Layer thickness			
Mucosa	1.8 ± 0.4 [1.2–2.6] (Gladwin et al. 2014)	2.0 ± 0.4 [1.5–3.2] (Gladwin et al. 2014)	2.2 ± 0.5 [1.1–3.2] (Gladwin et al. 2014)
Submucosa	0.5 ± 0.1 [0.3–0.9] (Gladwin et al. 2014)	0.6 ± 0.2 [0.3–1.0] (Gladwin et al. 2014)	0.6 ± 0.1 [0.3–0.8] (Gladwin et al. 2014)
Muscularis	0.5 ± 0.1 [0.2–0.7] (Gladwin et al. 2014)	0.5 ± 0.1 [0.3–0.8] (Gladwin et al. 2014)	0.5 ± 0.2 [0.3–0.9] (Gladwin et al. 2014)
Serosa	0.4 ± 0.1 [0.2–0.6] (Gladwin et al. 2014)	0.4 ± 0.1 [0.3–0.6] (Gladwin et al. 2014)	0.4 ± 0.1 [0.3–0.6] (Gladwin et al. 2014)
Ileum			
Full thickness	4.83 ± 1.22 [2.51–7.18] (Le Roux et al. 2016)		
Layer thickness			
Mucosa	2.84 ± 0.94 [1.31–4.58] (Le Roux et al. 2016)		
Submucosa	0.33 ± 0.1 [0.23–0.69] (Le Roux et al. 2016)		
Muscularis	1.13 ± 0.63 [0.12–2.71] (Le Roux et al. 2016)		
Inner muscularis	0.83 ± 0.48 [0.36–2.15] (Le Roux et al. 2016)		
Outer muscularis	0.35 ± 0.11 [0.2–0.56] (Le Roux et al. 2016)		
Serosa	0.19 ± 0.06 [0.07–0.33] (Le Roux et al. 2016)		
Colon			
Full thickness	1.5 ± 0.3 [1.0–2.0] (Gladwin et al. 2014)	1.4 ± 0.5 [1.1–1.9] (Gladwin et al. 2014)	1.6 ± 0.4 [1.1–2.6] (Gladwin et al. 2014)
Layer thickness			
Mucosa	0.4 ± 0.1 [0.2–0.6] (Gladwin et al. 2014)	0.4 ± 0.1 [0.2–0.5] (Gladwin et al. 2014)	0.5 ± 0.1 [0.3–0.7] (Gladwin et al. 2014)
Submucosa	0.4 ± 0.1 [0.2–0.6] (Gladwin et al. 2014)	0.3 ± 0.1 [0.2–0.4] (Gladwin et al. 2014)	0.4 ± 0.1 [0.2–0.5] (Gladwin et al. 2014)
Muscularis	0.4 ± 0.1 [0.2–0.7] (Gladwin et al. 2014)	0.3 ± 0.1 [0.2–0.5] (Gladwin et al. 2014)	0.4 ± 0.1 [0.2–0.7] (Gladwin et al. 2014)
Serosa	0.4 ± 0.1 [0.2–0.5] (Gladwin et al. 2014)	0.4 ± 0.1 [0.2–0.5] (Gladwin et al. 2014)	0.4 ± 0.1 [0.2–0.5] (Gladwin et al. 2014)

**Table 4.** Range of normal gastric and intestinal segment full-wall and individual layers thickness in puppies, according to the literature reviewed.

GI segment	Puppies wall range (mm)
Stomach full thickness	2.7 ± 0.4 [2.2–3.7] (Stander et al., 2010) 2.09 ± 0.05 (4w), 2.51 ± 0.04 (8w), 2.66 ± 0.1 (16w) (Banzato et al. 2017)
Duodenum full thickness	3.8 ± 0.5 [3.2–4.8] (Stander et al., 2010)
Jejunum full thickness	2.5 ± 0.5 [1.2–3.4] (Stander et al., 2010) 2.44 ± 0.04(4w), 2.73 ± 0.04 (8w), 2.78 ± 0.08 (16w) (Banzato et al. 2017)
Jejunum′s layer thickness	
Mucosa	1.48 ± 0.29 (4w), 1.76 ± 0.32 (8w) 1.99 ± 0.38 (16w) (Banzato et al. 2017)
Submucosa	0.36 ± 0.06 (4w), 0.40 ± 0.05 (8w), 0.47 ± 0.10 (16w) (Banzato et al. 2017)
Muscularis	0.13 ± 0.05 (4w), 0.17 ± 0.06 (8w), 0.26 ± 0.07 (16w) (Banzato et al. 2017)
Serosa	0.35 ± 0.05 (4w), 0.39 ± 0.05 (8w), 0.39 ± 0.06 (16w) (Banzato et al. 2017)
Colon full thickness	1.3 ± 0.3 [0.7–2.0] (Stander et al., 2010) 1.08 ± 0.03 (4w), 1.22 ± 0.02 (8w), 1.21 ± 0.05 (16w) (Stander et al., 2010)
Colon′s layer thickness	
Mucosa	0.20 ± 0.06 (4w), 0.26 ± 0.10 (8w), 0.25 ± 0.08 (16w) (Banzato et al. 2017)
Submucosa	0.41 ± 0.11 (4w), 0.44 ± 0.09 (8w), 0.49 ± 0.12 (16w) (Banzato et al. 2017)
Muscularis	0.11 ± 0.03 (4w), 0.16 ± 0.10 (8w), 0.17 ± 0.07 (16w) (Banzato et al. 2017)
Serosa	0.32 ± 0.05 (4w), 0.35 ± 0.06 (8w), 0.36 ± 0.06 (16w) (Banzato et al. 2017)

**W-**weeks.

**Table 5.** Range of normal gastric and intestinal segment full-wall and individual layers thickness in adult cats, according to the literature reviewed.

GI segment	Cat wall range (mm)
Stomach full thickness	Inter-rugal 2.03 [1.1–3.6] (Newell et al. 1999) Rugal fold: 4.38 [2.6–7] (Newell et al. 1999)
Fundus	2 [1.7–2.2], 95% CI (Goggin et al. 2000)
	0.19 [0.16–0.2] (Winter et al. 2014)
Body	0.22 [0.19–0.26] (Winter et al. 2014)
Pylorus	2.1 [1.9–2.4], 95% CI (Winter et al. 2014)
	0.21 [0.17–0.27] (Winter et al. 2014)
Stomach′s layer thickness	
Fundus	
Mucosa	0.12 [0.1–0.19] (Winter et al. 2014)
Submucosa	0.04 [0.03–0.05] (Winter et al. 2014)
Muscularis	0.06 [0.06–0.09] (Winter et al. 2014)
Serosa	0.03 [0.02–0.03] (Winter et al. 2014)
Body	
Mucosa	0.09 [0.06–0.11] (Winter et al. 2014)
Submucosa	0.04 [0.03–0.05] (Winter et al. 2014)
Muscularis	0.06 [0.05–0.08] (Winter et al. 2014)
Serosa	0.03 [0.03–0.04] (Winter et al. 2014)
Pylorus	
Mucosa	0.08 [0.06–0.10] (Winter et al. 2014)
Submucosa	0.04 [0.03–0.05] (Winter et al. 2014)
Muscularis	0.06 [0.04–0.08] (Winter et al. 2014)
Serosa	0.03 [0.02–0.03] (Winter et al. 2014)
Small intestine (no duodenum)	2.1 [1.6–3.6] (Newell et al. 1999)
Duodenum full thickness	2.2. [2–2.4] 95% Sedated 2.71 [1.6–3.5] (Newell et al. 1999)* Awake 2.4 [1.3–3.8] (Newell et al. 1999)*
Duodenum′s layer thickness	
Mucosa	0.15 [0.12–0.16] (Winter et al. 2014)
Submucosa	0.03 [0.03–0.04] (Winter et al. 2014)
Muscularis	0.04 [0.03–0.05] (Winter et al. 2014)
Serosa	0.03 [0.02–0.03] (Winter et al. 2014)
Jejunum full thickness	2.3 [2.1–2.5] 95% CI (Goggin et al. 2000) 2.22 [1.96–2.67] (Di Donato et al. 2014)
Jejunum′s layer thickness	
Mucosa	0.11 [0.10–0.14] (Winter et al. 2014)
Submucosa	0.03 [0.03–0.04] (Winter et al. 2014)
Muscularis	0.04 [0.03–0.07] (Winter et al. 2014)
Serosa	0.03 [0.02–0.03] (Winter et al. 2014)
Ileum full thickness	2.8 [2.5–3.2], 95% CI (Goggin et al. 2000) Fold 3 [2.52–3.59] (Di Donato et al. 2014). Between folds 2 [1.66–2.27] (Di Donato et al. 2014)
Ileum′s layer thickness	
Mucosa	0.12 [0.09–0.15] (Winter et al. 2014)
Submucosa	0.03 [0.03–0.05] (Winter et al. 2014)
Muscularis	0.08 [0.06–0.1] (Winter et al. 2014)
Serosa	0.03 [0.03–0.03] (Winter et al. 2014)
Colon full thickness	1.5 [1.4–1.7], 95% CI (Di Donato et al. 2014) 1.67 [1.1–2.5] (Newell et al. 1999)
Colon′s layer thickness	
Mucosa	0.04 [0.04–0.05] (Winter et al. 2014)
Submucosa	0.03 [0.02–0.03] (Winter et al. 2014)
Muscularis	0.03 [0.02–0.03] (Winter et al. 2014)
Serosa	0.02 [0.02–0.03] (Winter et al. 2014)

CI: Confidence interval.

* Sedation evidenced a significant effect on duodenal wall thickness.

To provide a transparent, study-level assessment of methodological quality we applied the AXIS (Appraisal tool for Cross-Sectional Studies) checklist to each included study (Downes et al. 2016). Two reviewers (MEM and CSB) independently scored all 20 AXIS items as Yes/Partial/No/Unclear; disagreements were resolved by discussion and, where necessary, by consensus with a third reviewer (MDA). The full item-by-item matrix (20 items × 12 studies) and the brief justifications used for each judgment are provided as Supplementary Tables S1 and S2. Summary counts of AXIS judgments are presented in the Results. The AXIS assessment informed the qualitative synthesis and the interpretation of the proposed reference intervals.

**Table 1.** Analysis of the publications regarding US evaluation of the normal GI tract in cats.

Study	Animals	Variables / measurements	Normal GI status	Conclusions	Limitations of the study
Newell et al. (1999**)**	*n =* 14 1–9 years No gender No weight Alive	1. Full wall thickness. 2. Stomach (rugal, inter-rugal), proximal duodenum, small intestine, descending colon. 3. Three levels of stomach distension. 4. Awake and sedated (ketamine hydrochloride + acepromazine maleate). 5. Three measurements *per* anatomic region (average). 6. Sagittal and transverse plans. 7. 10 MHz Probe.	Histopathology Physical and laboratory evaluation (CBC, serum chemistry, urianalysis, fecal examination)	1. Sedation increases full thickness of the duodenum as a single factor but did not significantly affect any of the parameters measured. 2. The thickness of the rugal folds was significantly higher than the thickness of the inter-rugal regions, meaning that separate standards of normal thickness should be recognized for these two functional areas of the stomach. 3. Distension of the stomach does not significantly change the thickness of the rugal folds or inter-rugal regions. 4. Reports full wall normal thickness of stomach (rugal and inter-rugal foals), proximal duodenum, small intestine, descending colon.	1. Two cats (2/14) had mild histologic evidence of colitis with variable inflammatory cells, although there was no destruction or alteration of the normal histologic architecture of the colon (not known if these cats presented larger measurements for the colon). 2. Not all variables were assessed for each cat, not allowing individual variations according to distension and sedation status. 3. No differentiation of gastric segments (fundus, pylorus), neither jejunum nor ileum (overall small bowel). 4. Limited statistical value (relative low numbers). 5. Unknown gender.
Goggin et al. (2000**)**	*n =* 11, initially *n =* 9, after histopathology No age 4 females Normal body condition Alive	1. Full wall thickness. 2. Gastric fundus, pylorus, duodenum, jejunum, ileum, transverse colon. US features of the ileocolic region. 3. Twelve hours fasting. 4. General anesthesia with (halothane). 5. Three measurements *per* anatomic region (average). 6. Cross-sectional images. 7. 10 MHz Probe.	Histopathology Not determined clinically	1. Reports on the appearance of the ileum. 2. Establishes comparisons with dog, equine and humans. 3. Reports full wall normal thickness of stomach (inter-rugal folds), duodenum, jejunum, ileum, and transverse colon.	1. No comparison with Newell et al. 1999. 2. No assessment of stomach distension. 3. No measurements of stomach rugal folds. 4. Unknown influence of anesthesia with halothane in GI tract wall thickness. 5. Results with limited statistical value (low numbers, nonuniform distribution of males and females; slightly oblique positioning which may affect significantly thinner colon and significantly thicker ileum results). 6. Unknown age.
Winter et al. (2014**)**	*n =* 38 0.5 to 16 years No gender 5.2 ± 1.5 kg Alive	1. Muscularis, submucosal, mucosal and serosal layers thickness of the GI wall. 2. Gastric fundus, body and pyloric antrum, duodenum, jejunum, ileum, colon. 3. Determines the ratio of muscularis (Musc:Ao) and mucosal (Muc:Ao) layer thickness to aortic diameter measured at the level of the celiac artery. 4. Establishes the type of food ingested (wet and/or dry). 5. Twelve hours fasting. 6. Sedated with ketamine hydrochloride (4 mg/kg, IV) and diazepam (0.2 mg/kg IV). 7. Three measurements per each layer (average). 8. Transverse plan. 9. 5–17 MHz Probes.	No histopathology Without clinical evidence of GI disease (weight loss, inappetence, vomiting, previous diagnoses of neoplasia) or evidence of other chronic disease Complete blood count and serum biochemical analysis	1. Reports baseline layer thickness measurements in each segment of the GI tract. 2. Musc:Ao and Muc:Ao ratios are clinically relevant values that can be used to objectively identify thickening of the muscularis and mucosal layers. 3. There was no correlation between age and GI layer thickness. 4. Cats fed with a combination of wet and dry food had a small, but significantly greater, mucosal layer thickness in the ileum than cats fed with dry food only.	1. Some of the measurements made were at the limits of the spatial resolution of the transducer, specifically those of the serosal and submucosal layers, and the accuracy of these extremely small measurements can be questioned. 2. Diet was not a controlled variable in the study. Including more cats on a wet food diet alone, or on different diet formulations, may reveal differences in layer thicknesses. 3. There was no confirmation of the normal GI trat status with full-thickness intestinal biopsies.
Di Donato et al. (2014)	*n =* 20 (8 intact males, 6 neutered males, 1 intact female and 5 neutered females) 4.4 ± 0.9 kg (3 to 6 kg) 1 to 7 years (mean: 3.4 ± 2 years)	1. Jejunal, duodenal, ileal (fold and between folds) and duodenal images. 2. Measurements of full thickness wall, mucosal, submucosal, muscular and serosal thickness (consecutive measurements of each of the layer). 3. Awake and restrained manually during the examination. 4. Twelve hours fasting. 5. Longitudinal and transverse (transverse was used for colon measurements). 6. 13 MHz Probe.	Physical examination, complete blood count, routine serum biochemical analyses, urinalysis and faecal examination for intestinal parasites	1. The thickness of ileum at the level of the fold was significantly higher than the other intestinal segments. 2. The relative proportion values provided in this study can be useful as a baseline reference when evaluating feline intestinal disorders, such as inflammatory bowel disease and round cell tumours, that can have different degrees of intestinal layer involvement.	1. The population of cats prospectively recruited was considered healthy only on the basis of clinical and laboratory findings. No endoscopic or surgical biopsies were taken and, therefore, histological confirmation of the absolute normality of the intestinal wall was not available. 2. The measurements of each individual layer of feline intestinal wall could be influenced by observer’s experience, and the evaluation of inter-observer variability was not performed in this study. 3. The accuracy of the thickness of layers of ileum can be questioned owing to the lower number of these measurements and to the difficulty to clearly distinguish the layers for the presence of folds. Therefore, these values should be validated in a wider feline population.
Hahn et al. (2017**)**	*n =* 20 (4 females, 16 male) 4.3 kg (3.8–4.6) 1–8 years (average of 3) Alive	1. Ultrasound appearance of the proximal and distal caecum in the asymptomatic adult cat. 2. Correlation to endoscopic and histological findings. 3. General anesthesia (induction with diazepam and propofol and then the cats were intubated with isoflurane). 4. Twelve hours fasting. 5. Longitudinal. 6. 18 MHz Probe.	History (no clinical evidence of gastrointestinal disease: no diarrhea, hematochezia, weight loss, vomiting or dyspraxia during the last 3 months) and physical examination	1. Subclinical mild caecal inflammation could be found in asymptomatic cats. 2. Among all measured US parameters, the most accurate one in detecting this subclinical state was the thickness of the caecal folicular layer. 3. Ultrasonography had a higher agreement with histology than with endoscopy in the evaluation of a mildly inflamed caecum. 4. The agreement between caecal and colonic inflammation among a single evaluation technique was unsatisfactory for all three techniques. Ultrasonography, endoscopy and histopathology are complementary to evaluate the caecum fully.	1. The sample of cats was relatively small and there was a majority of Siamese intact male cats, which is not representative of the feline population. The low number of cats may have led to imprecise estimations of the κ values between histological, endoscopic and US results.
Martinez et al. (2018**)**	*n =* 17 (6 adult males and 11 adult females) 3.6 kg (2.8–8.6 kg) Young adults Cadavers (euthanized for reasons unrelated to gastrointestinal tract disorders, such as behavioral or orthopedic problems)	1. Full thickness wall and measurements of each layer (mucosa, submucosa, muscularis, and serosa) for each segment of the small intestine (duodenum, jejunum and ileum). 2. Three measurements *per* anatomic region. 3. Relationship between ultrasonographic measurements in the transverse and longitudinal planes. 4. Relationship between ultrasonographic and histologic thickness. 5. Cadavers. 6. Twelve hours fasting. 7. Longitudinal. 8. 13 MHz Probe.	Not described (but none of the cats’ showed signs of GI tract disease, which was supported on histopathologic evaluation by a lack of abnormal cellular infiltrates or other.	1. There were no significant differences between ultrasonographic measurements in longitudinal and transverse planes of intestinal specimens, except for the distal ileum at the level of the fold. 2. There was good agreement between ultrasonographic and histologic measurements of the total wall thickness and the layers of the different intestinal segments, except at the submucosa and muscularis of the duodenum.	1. Extensive medical history, hematology, biochemistry, urinalysis or faecal examination, were not available for the cats studied. 2. The US transducer resolution was not confirmed experimentally. 3. The time between euthanasia and fixation of intestinal samples was approximately 1 h (a study in rats reported changes in the intestinal mucosa 40 min post-mortem due to dehydration, which induced a loss of turgidity and stiffness and a thinning of the intestinal villi). 4. The ideal fixation time in formalin was not established and standardized for the tissues obtained and it is unclear whether different fixation times could alter the histological thickness of the samples.

CBC: Complete blood count.

**Table 2.** Analysis of the publications regarding US evaluation of the normal GI tract in dogs.

Study	Animals	Variables / measurements	Normal GI status	Conclusions	Limitations of the study
Penninck et al. (1989**)**	Group 1–4 Beagles; Group 2–4 small dogs; Group 3–4 large dogs; Group 1 – 8–12 kg; Group 2 - 4–6 kg; Group 3 - 32–54 kg; Group 1 – US performed at 13, 27, and 40 weeks of age	1. Appearance and mural thickness transverse and longitudinal images of the stomach, proximal duodenum, small bowel and descending colon. 2. Evaluation of degree of distension. 3. Awake. 4. Transverse and longitudinal. 5. 5.0-MHz and/or a 7.5-MHz Probe.	Physical examination, laboratory work (CBC, serum chemistry, fecal examination), and lack of historical information concerning GI disease	1. Standardization of the GI wall thickness measurement is possible by imaging the wall during bowel relaxation. 2. Submucosa and subserosa/ serosa are hyperechoic due to the presence of relatively more fibrous connective tissue. 3. No significant difference was noted between the GI wall thickness of immature and mature dogs of the same breed, nor between dogs of small, medium, and large breeds.	1. The small number of dogs in each category is a limiting factor for any conclusive statistical analysis. The author suggested that the measurements presented the paper should be considered only as a starting point for further investigation. 2. Colonic measurements were often compromised by large amounts of intraluminal gas.
Delaney et al. (2003**)**	*n =* 231 (gender was not specified) From 2.1 to 64.0 kg (median 23.0 kg)	1. Two measurements of jejunum and 1 of duodenum. 2. Dogs placed into one of five groups based on body weight. 3. Awake. 4. Longitudinal. 5. 7.5 to 11 MHz Probes.	Physical exam and clinical history	1. Overall, the wall thickness of the duodenum was significantly greater than that of the jejunum; 2. As weight increased, a significant increase in duodenal wall thickness was also observed, but not in the jejunum.	1. No description of sex and age. 2. Duodenum was not clearly identified in all dogs.
Stander et al. (2010**)**	*n =* 23 (8 females and 15 males) 2.3 to 5 kg (3.0 ± 0.7 kg) 7 to 12 weeks of age (mean 8.8 ± 1.8 weeks)	1. Stomach wall measurements between rugal folds 2. Intestinal wall measurements: a single measurement of the proximal descending duodenum and 2 measurements of the jejunum. 3. Descending colon 4. Width and echogenicity of the jejunal and duodenal mucosa. 5. Wall layering. 6. Awake. 7. Sagittal and transverse. 8. 7.5–9 MHz Probes.	Physical and laboratory evaluation (peripheral blood smear evaluation and fecal analysis)	1. Wall thickness of the gastrointestinal segments relative to each other appears to follow similar trends in puppies vs. adults. There was no significant effect of age or weight on jejunal or colonic wall thickness and on jejunal or duodenal mucosal thicknesses. 2. There was a significant increase in duodenal and stomach wall thickness with increase in age and weight. 3. Duodenal and jejunal mucosal layers are the thickest of the wall layers. The mean duodenal mucosal thickness constituted 71% of the total wall thickness and the mean jejunal mucosal thickness 60%. 4. In the stomach the mucosa, submucosa, and muscularis were of equal thickness, but with a thinner serosa. 5. All colonic wall layers appeared to have a comparable thickness.	1. Ultrasonography of canine pediatric patients was challenging due to poor compliance. They were difficult to restrain and reluctant to lie stationary for the 20 min ultrasonographic examination. The noncompliance led to progressive aerophagia, which hampered assessment of some structures. 2. Stomach and colonic wall measurements were not obtained in all the puppies due to noncompliance.
Gladwin et al. (2014**)**	*n =* 85 (gender was not specified) Adults (>12months)	1. Images of duodenum, jejunum and descending portion of colon. 2. Measurements were obtained from a single transverse image of the duodenum, jejunum, and colon and included total wall thickness and thickness of the mucosa, submucosa, muscularis, and serosa. 3. Dogs were placed into one of three groups based on body weight. 4. Awake. 5. Transverse.	Physical exam and clinical history: no signs of gastrointestinal tract disease (vomiting, diarrhea, anorexia, or weight loss) during the 2 months preceding the abdominal	1. The mucosal layer was the thickest layer of the duodenum and jejunum. 2. There was a significant difference in thickness of the mucosal layer between small and large dogs.	1. Histologic examination was not performed to confirm a lack of abnormalities in the gastrointestinal tract of each dog. 2. Small sample size in each group, which may have affected the statistical power of the reference values. 3. All measurements were made by only 1 investigator who used still images to standardize the way in which measurements were obtained. This study design did not address interobserver variation.
Le Roux et al. (2016)	*n =* 12 (6 males and 6 females) Average weight of 23.4 ± 5.2 kg (4 dogs were between 10 and 20 kg, 6 between 20 and 30 kg, and 2 between 30 and 40 kg) Assumed to be young adults	1. Measurement of mid-segments of duodenum, jejunum and ileum. 2. Three measurements performed independently for each intestinal layer. 3. Dead. 4. Histopathological correlation. 5. Transverse. 6. 13 MHz (for total wall thickness) and 15 MHz Probes (for total wall thickness and individual layers, to correlate with the histopathology).	Not described	1. There were significant statistical differences between histological and ultrasonographic layer thicknesses in the small intestine of adult dogs. 2. Strong to very strong positive correlation between ultrasonographic and histological layer thickness, except for the serosa.	1. Experimental evaluation of the ultrasound transducer resolution was not assessed. The image resolution of post processing image viewer application was approximately 0.06 mm. Therefore, for small measurements close to the axial and imaging software resolution, such as the serosa, this could have been a potential source of measurement errors. 2. Some of the ultrasonographic and histological small intestinal measurements appear abnormally thicker in comparison to normal reported values in dogs. The values provided should however not be interpreted on their own, but only as a comparison between the two modalities used to assess intestinal layer correlation (ultrasonography and histology). 3. Direct comparison of intestinal wall thickness measurements between *in vivo* and *ex vivo* intestinal segments will likely be inaccurate, as the length and thickness of a resection specimen can change after devitalization, formalin fixation, and histological section.
Banzato et al. (2017**)**	*n =* 84 (gender was not specified) At 4 weeks: 2.2 ± 0.7 kg (ranging from 1 to 3.6); at 8 weeks was 4.3 ± 1.8 kg (ranging from 1.5 to 8.5); at 16 weeks: 9.8 ± 5.4 kg (ranging from 3.5 to 19.9). 4, 8 and 16 weeks of age	1. Measurement of stomach, duodenum, jejunum and colon performed at each age in every puppy. 2. Awake. 3. Transverse. 4. 8–12 MHz Probes.	Physical evaluation, body weight and body condition score	1. Increase in the wall thickness of all the gastrointestinal tracts during development; the effect of age was stronger on the stomach, duodenal and jejunal wall thicknesses and lesser on the colonic wall thickness. The effect of body weight was marked on duodenal and jejunal wall thicknesses whereas a lower effect of body weight on stomach and colonic wall thickness was evident. 2. Body weight can be used to determine the expected duodenal and jejunal wall thicknesses in developing puppies between four and sixteen weeks of age. 3. Strong positive correlation resulted evident between duodenal and jejunal mucosal layer thicknesses and body weight, whereas the correlation between body weight and the remaining intestinal layers of the duodenum, jejunum and colon ranged from moderate to weak.	1. The individual stomach wall layers were not always clearly identified and therefore only wall thickness was measured. No measurements for the ileum are reported. 2. No gender differentiation.

Due to the marked methodological heterogeneity among studies—including differences in ultrasound equipment, transducer frequency, scanning protocols, animal positioning, fasting status, and population characteristics—a quantitative meta-analysis was not performed, as pooled effect estimates would be unreliable and potentially misleading. Therefore, a structured descriptive synthesis of the available data was performed and presented in Tables 3–5.

The design and writing of this review was based on the guidelines recommended in the Preferred Reporting Items for Systematic Reviews and Meta-Analyses (PRISMA) statement (Page et al. 2021).

## 3. Results

**Figure 1.**
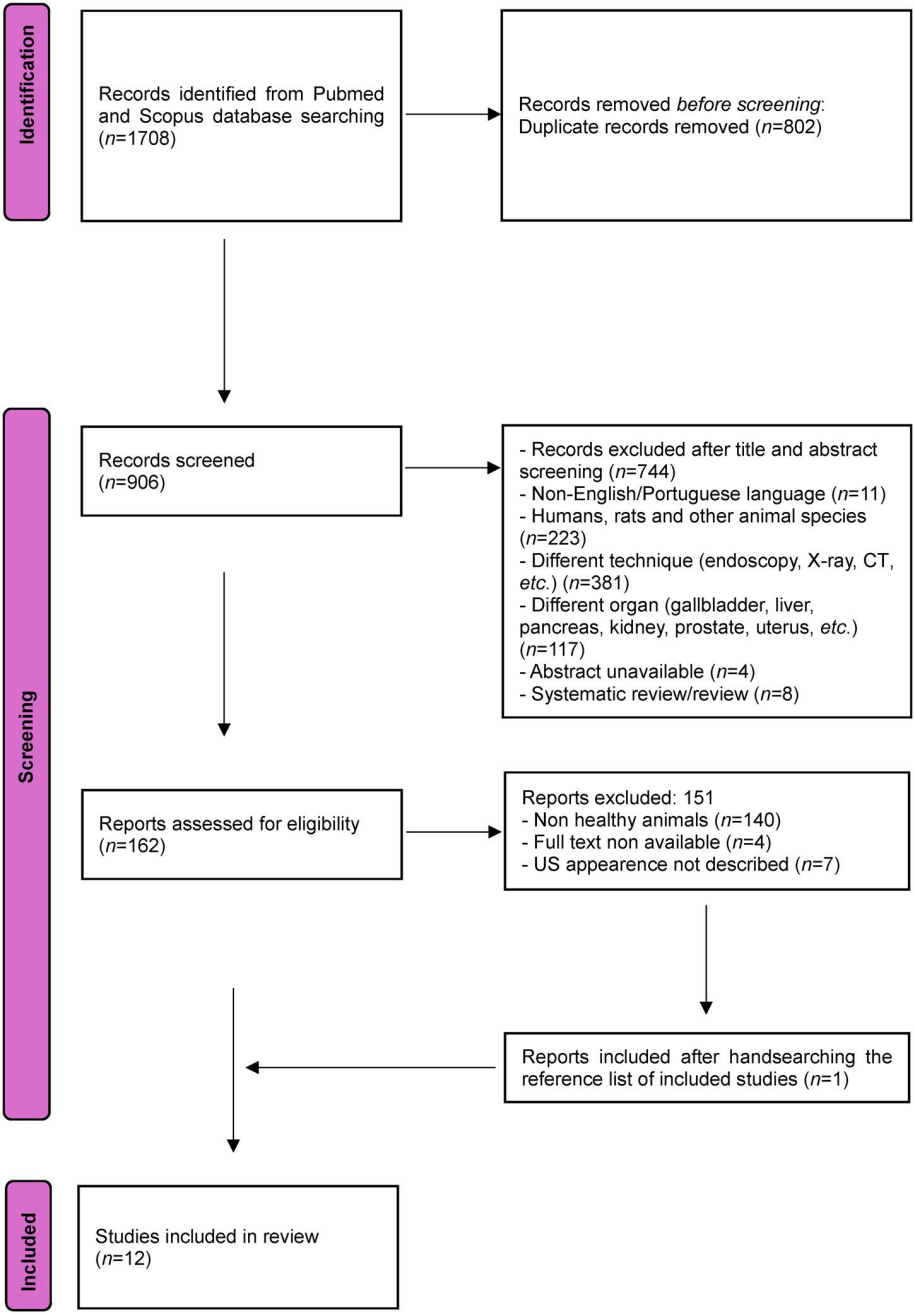
PRISMA flowchart of selected studies.

A total of 1708 results were identified through searches in the PubMed and Scopus databases, and their respective titles and abstracts were carefully reviewed. After applying inclusion and exclusion criteria, 1546 studies were deemed ineligible, leaving 162 for a comprehensive full-text assessment. Out of these, 102 papers were excluded for not meeting the inclusion criteria. The remaining papers were categorized into two groups: those related to the evaluation of normal GI US and those concerning pathological evaluation. Among these, 11 papers fulfilled all the inclusion criteria for this paper. Additionally, one relevant manuscript was discovered by consulting the reference lists of the initially selected papers. Consequently, a total of 12 papers were included in the final review (see Figure 1), and data from these papers were collected and summarized (Table 1 and 2).

### 3.1. Study population

Out of the 12 papers that examined the typical US appearance of the GI tract in small animals, six were dedicated on cats, including study population ranging from 9 to 38 individuals (Newell et al. 1999; Goggin et al. 2000; Di Donato et al. 2014; Winter et al. 2014; Hahn et al. 2017; Martinez et al. 2018), while the remaining six investigated dogs, with sample size ranging from 12 to 231 (Penninck et al. 1989; Delaney et al. 2003; Stander et al., 2010; Gladwin et al. 2014; Le Roux et al. 2016; Banzato et al. 2017).

In most of these papers, males were either the predominant subjects or their gender was not specified (Penninck et al. 1989; Newell et al. 1999; Delaney et al. 2003; Winter et al. 2014; Banzato et al. 2017). Among the papers discussing cats, they predominantly studied young adults to adults as subjects, while two of the papers discussing dogs were focused on puppies (Stander et al., 2010; Banzato et al. 2017), one on puppies and adults (Penninck et al. 1989), with the remainder dedicated to young adults to adults. Two papers did not specify an age group at all (Goggin et al. 2000; Delaney et al. 2003), and other two only assumed a possible age based on dentition (Le Roux et al. 2016; Martinez et al. 2018). None of the papers investigated the GI tract of older animals. One of the cat-focused papers did not mention the weight of the subjects (Newell et al. 1999), while other specified a ‘normal body condition’ (Goggin et al. 2000)^.^ The weights of the remaining cats ranged from 3.6 to 5.2 kg; however, the results were not categorized based on different weight ranges. Only one of the studies on dogs did not report the animals’ weight, however dogs were still divided into groups based on weight (Gladwin et al. 2014). The papers that studied puppies evaluated the GI tract according to age and weight (Penninck et al. 1989; Stander et al., 2010; Banzato et al. 2017). Three papers categorized the results based on different weight intervals (Penninck et al. 1989; Delaney et al. 2003; Gladwin et al. 2014), while the last did not categorize by weight since the focus was to correlate with histopathology findings (Le Roux et al. 2016).

All dogs were alive and healthy when examined, except in one case where the animals were euthanized for reasons unrelated to GI disease (Le Roux et al. 2016). All cats were also alive during the US except for one study where they used cadavers euthanized for reasons unrelated to GI tract disorders, such as behavioral or orthopedic problems (Martinez et al. 2018). In another paper, the cats were euthanized immediately after imaging (Goggin et al. 2000). Most of the authors considered the absence of GI disease based on physical exams and clinical history. Only two papers did not describe any assessment of normal GI tract status (Goggin et al. 2000; Le Roux et al. 2016).

### 3.2. Study design

All studies are observational. All dogs were awake during examination and were physically restrained (except the ones that were euthanized (Le Roux et al. 2016)). Regarding cats, only one study kept the animals awake and restrained manually during the examination (Di Donato et al. 2014), while the remaining authors either sedated the cats or put them under general anesthesia.

Only two studies measured the degree of distension of the stomach (Penninck et al. 1989; Newell et al. 1999) and histopathology was performed in six studies (Penninck et al. 1989; Newell et al. 1999; Goggin et al. 2000; Le Roux et al. 2016; Hahn et al. 2017; Martinez et al. 2018). The frequency of the probe was not specified in one of the studies (Gladwin et al. 2014) and varied significantly among the remaining papers, ranging from 5 to 18 MHz. Higher probe frequencies were generally used in cat studies. The imaging plane also varied among the studies, with the transverse plane present in all except one (Hahn et al. 2017).

In the cat studies, several variables were measured, including: 1) full thickness of stomach (Newell et al. 1999,Winter et al. 2014; Goggin et al. 2000); 2) thickness of the individual layers of the stomach wall (Winter et al. 2014); 3) full thickness of small intestine (Newell et al. 1999; Di Donato et al. 2014; Winter et al. 2014; Martinez et al. 2018,Goggin et al. 2000); 4) thickness of the individual layers of the small intestine wall (Di Donato et al. 2014; Winter et al. 2014; Martinez et al. 2018); 5) ileocolic region (Goggin et al. 2000); 6) cecum thickness and layering (Hahn et al. 2017); 7) full thickness of colon (Newell et al. 1999; Goggin et al. 2000; Winter et al. 2014; Hahn et al. 2017); 8) thickness of the individual layers of colonic wall (Winter et al. 2014). In three studies, the variables measured were full thickness of stomach, small bowel and colon (Penninck et al. 1989; Stander et al., 2010; Banzato et al. 2017), and thickness of the individual layers in the same portions (Banzato et al. 2017), correlating with age and body weight. The US image of the wall layering was also described (Penninck et al. 1989; Stander et al., 2010).

In the adult dog studies, the variables measured were: 1) full thickness of the small intestine (Delaney et al. 2003; Gladwin et al. 2014; Le Roux et al. 2016); 2) thickness of the individual wall layers of the small intestine (Gladwin et al. 2014; Le Roux et al. 2016); 3) full thickness and individual layers of colonic wall (Gladwin et al. 2014).

### 3.3. Imaging outcomes

**Figure 2.**
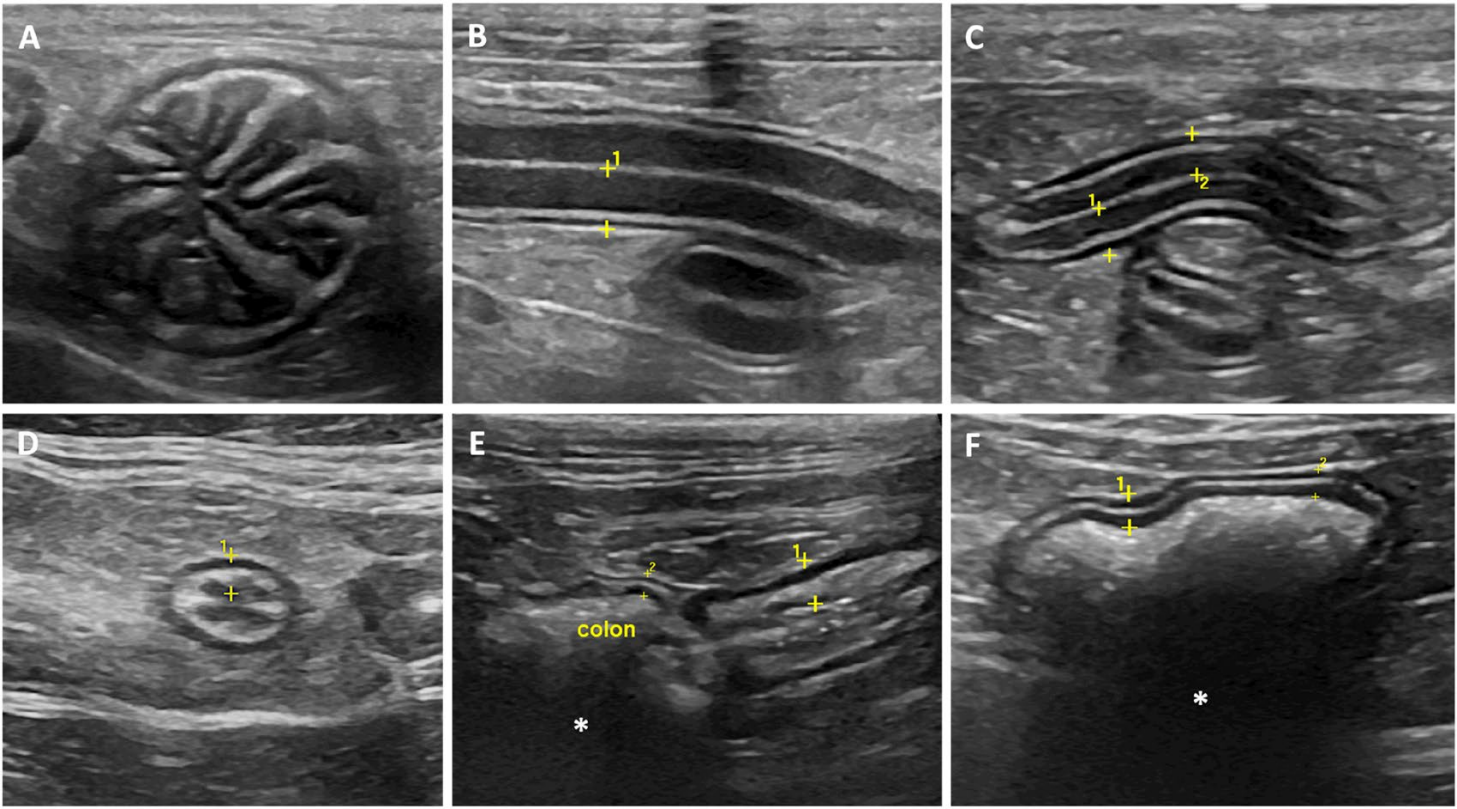
Ultrasonographic images of the gastrointestinal segments routinely assessed in the US evaluation of dogs and cats (linear probe 9–11 MHz). (A) short axis US image of an empty stomach of a cat (rugal folds); (B) Longitudinal US image of the duodenum of a dog, and (C) jejunum of a cat, evidencing the five echogenic layers; (D) Transverse US image of the distal ileum of a cat; (E) Ileocecocolic region, cat: ileum and gas filled colon, exhibiting acoustic shadowing (*); (F) Colon, dog: gas filled, evidencing acoustic shadowing (*). Full wall between clippers in all images.

On US, intestinal sections present a five-layered appearance with alternating hyper- and hypoechoic layers, corresponding to the mucosal surface, mucosa, submucosa, muscularis, and serosa, respectively (Penninck et al. 1989). Five echogenic layers were identified: the innermost hyperechoic layer corresponds to the surface of the mucosa; the innermost hypoechoic layer represents the mucosa; the mid hyperechoic layer is the submucosa; the outer hypoechoic layer is the muscularis propria; and the outer hyperechoic layer is the subserosa/serosa (Penninck et al. 1989). Figure 2 presents representative images of normal gastrointestinal (GI) segments obtained with the equipment General Electric Logiq S8 R3 XDclear, linear probe 9–11 MHz, of dogs and cats to illustrate the ultrasonographic features described in the reviewed studies. These images are for illustration only and were not included in the data analysis.

In the studies including dogs (*n* = 6), two presented the results categorized by the weight of the animals (*n* = 2/6) (Delaney et al. 2003; Gladwin et al. 2014), one by the age (*n* = 1/6) (Banzato et al. 2017), and another one divided by weight and age (*n* = 1/6) (Penninck et al. 1989). The stomach was evaluated in the three papers (*n* = 3/6) that conducted US scans on puppies (Stander et al., 2010; Banzato et al. 2017) and in one paper that studied puppies and adults (Penninck et al. 1989). In this last study, there was no significant difference between the gastric wall thickness of immature and mature dogs of the same breed, nor between dogs of small, medium, and large breeds (Penninck et al. 1989). Small and medium adult dogs presented gastric wall thickness of 3 mm (Penninck et al. 1989) (Table 3). In the studies focused only on puppies, the mean gastric thickness ranges from 2.09 mm in 4 weeks old puppies to 2.66 mm, at 16 weeks (Banzato et al. 2017) (Table 4).

Regarding the small intestine and colon, the most recent study categorizes dogs by weight into three groups: <15 kg, 15–30 kg, and >30 kg (Gladwin et al. 2014). The maximal mean wall thickness of duodenum (in mm) is 3.8 (< 15 kg), 4.1 (15 to 30 kg) and 4.4 (> 30 kg). The maximal mean wall thickness of jejunum (in mm) is 3.0 (< 15 kg), 3.5 (15 to 30 kg) and 3.8 (> 30 kg) (Gladwin et al. 2014). However, ileum was only evaluated in one study where the mean wall thickness was 4.83 mm (Le Roux et al. 2016). The maximal mean wall thickness of colon (in mm) is 1.5 (< 15 kg), 1.4 (15 to 30 kg) and 1.6 (> 30 kg) (Table 3).

In cats (Table 5), the gastric wall was evaluated in the fundus, body and pylorus areas. The maximal mean values of the gastric body at the level of the rugal fold can reach up to 4.38, and 2.03 mm for the inter-rugal space, with no differences according to the degree of stomach distension (Newell et al. 1999). The mean thickness of the gastric fundus (between rugal folds) was 2.0 mm, and the pylorus mean thickness (between rugal folds) was 2.1 mm (Goggin et al. 2000). The maximal mean wall thickness described for the duodenum was 2.47 mm (Martinez et al. 2018), for jejunum was 2.3 mm (Goggin et al. 2000), and for ileum was 3 mm (Di Donato et al. 2014). The ileocolic region presented a characteristic ‘wagon wheel’ appearance on cross-sectional images; significantly thicker than all other portions of the GI tract (Goggin et al. 2000). The maximal cecal wall thickness was 3.1 mm (Hahn et al. 2017), and the maximal mean colonic wall thickness was 1.67 mm (Newell et al. 1999) (Table 5 and Figure 2).

### 3.4. Main observations

In the papers focused on cats, one of the conclusions was that sedation, as a single factor, does not significantly affect any of the parameters measured of the GI tract wall thickness (Newell et al. 1999). Also, it seems that there is no correlation between age and GI tract layer thickness (Winter et al. 2014) and that there are no significant differences between ultrasonographic measurements in longitudinal and transverse planes (Martinez et al. 2018). Regarding the stomach, the thickness of the rugal folds is significantly higher than the thickness of the inter-rugal regions, meaning that separate standards of normal thickness should be recognized for these two functional areas of the stomach. Also, distension of the stomach does not significantly change the thickness of the rugal folds or inter-rugal regions (Newell et al. 1999).

In dogs, studies with puppies came to conflicting conclusions. The first study found no significant effect of age or weight on jejunal or colonic wall thickness but observed a significant increase in duodenal and stomach wall thickness with age and weight (Stander et al., 2010). However, another study reported an increase in the wall thickness of all the GI tracts during development. The effect of age was more pronounced on the stomach, duodenal, and jejunal wall thicknesses, and less pronounced on the colonic wall thickness, though it was still evident. Additionally, the impact of body weight was more significant on duodenal and jejunal wall thicknesses, while a lesser effect of body weight on stomach and colonic wall thickness was observed (Banzato et al. 2017). The third study concluded that there was significant difference between the GI wall thickness of immature and mature dogs (Penninck et al. 1989).

Overall, the wall thickness of the duodenum seems to be significantly greater than that of the jejunum (Delaney et al. 2003). As weight increased, a significant increase in duodenal wall thickness was also observed, but not in the jejunum (Delaney et al. 2003) nor stomach (Penninck et al. 1989).

### 3.5. Limitations

One of the first limitations described by the authors was the low numbers of animals, resulting in limited statistical value (Penninck et al. 1989; Newell et al. 1999; Goggin et al. 2000; Gladwin et al. 2014; Hahn et al. 2017). There are also some limitations regarding study population such as unknown age (Goggin et al. 2000; Delaney et al. 2003; Le Roux et al. 2016; Martinez et al. 2018) or gender (Penninck et al. 1989; Newell et al. 1999; Winter et al. 2014; Banzato et al. 2017). Half of the studies used animals that were considered healthy based solely on their history, clinical and laboratory findings (with no signs of GI disease). No endoscopic or surgical biopsies were taken; therefore, histological confirmation of the absolute normality of the intestinal wall was not available (Delaney et al. 2003; Stander et al., 2010; Di Donato et al. 2014; Gladwin et al. 2014; Winter et al. 2014; Banzato et al. 2017). Out of the 6 studies involving cats, in 4 of them, the animals were either sedated or under anesthesia. Therefore, there is an unknown influence of anesthesia/sedation on GI tract wall thickness (Newell et al. 1999; Goggin et al. 2000; Winter et al. 2014; Hahn et al. 2017). This was not a limitation in dog studies, as the animals were awake and physically restrained. However, in the puppy studies, noncompliance resulted in aerophagy, making it difficult to observe the stomach (Stander et al., 2010) and colonic wall (Penninck et al. 1989; Stander et al., 2010). Another limitation described was the difficulty to observe some structures such as stomach wall layers (Banzato et al. 2017), duodenum (Delaney et al. 2003) and ileum (Di Donato et al. 2014).

### 3.6. Risk-of-bias assessment (AXIS)

We applied the AXIS checklist to all 12 included studies (full matrix in Supplementary Tables S1 and S2). The distribution of overall appraisals was: Low risk—3 studies; Low–moderate risk—3 studies; Moderate risk—5 studies; High risk—1 study (Table 6; see Supplementary Tables S1 and S2 for study-by-study item scores and justifications). Common methodological shortcomings identified by AXIS were (1) absence of formal sample-size calculations, (2) scarce reporting of inter-/intra-observer reproducibility for ultrasound measures, (3) frequent use of convenience sampling from referral/academic centers, and (4) heterogeneity in probe frequency, fasting and sedation protocols. These issues lower the confidence in the precision and generalizability of the pooled descriptive intervals and were considered when drafting the consensus reference table (Table 6).

**Table 6.** Summary of the AXIS assessment for the 12 studies included in the review.

Study	Yes	Partial	No	Unclear	Overall appraisal (interpretation)
Newell et al. (1999)	8	8	3	1	**Moderate risk—**small N; limited reporting on sampling/interobserver.
Goggin et al. (2000)	8	8	3	1	**Moderate risk—**small N; anesthesia effect uncertain.
Winter et al. (2014)	13	5	2	0	**Low risk—**good layer detail and CIs but no histology; interobserver not reported.
Di Donato et al. (2014)	12	5	3	0	**Low-moderate risk—**clear methods; limited N; no interobserver stats.
Hahn et al. (2017)	12	4	4	0	**Moderate risk—**solid correlation but small N and breed-skewed (Siamese cats).
Martinez et al. (2018)	12	5	2	1	**Low-moderate risk—**useful histopathology comparison but *ex-vivo* limits *in vivo* generalizability
Penninck et al. (1989)	7	8	4	1	**High risk—**foundational but small groups and older reporting standards.
Delaney et al. (2003)	10	5	4	1	**Low-moderate risk—**strong N improves precision though some reporting gaps remain.
Stander et al. (2010)	9	6	4	1	**Moderate risk—**small pediatric sample; missing measurements due to aerophagia.
Gladwin et al. (2014)	12	4	3	1	**Low risk—**reasonable N but single-operator measurement and limited interobserver data.
Le Roux et al. (2016)	10	4	5	1	**Moderate risk—**strong methodology for correlation but ex-vivo caveats.
Banzato et al. (2017)	12	5	2	1	**Low risk—**good developmental coverage but technique/compliance limits.

For each study, the number of AXIS items scored as *yes*, *partial*, *No*, or *unclear* is reported. The overall appraisal represents a qualitative summary judgment based on the pattern of AXIS responses rather than on a numerical threshold. Color coding is used to facilitate visual interpretation of the overall risk-of-bias assessment. Detailed item-by-item AXIS evaluations and justifications are provided in Supplementary Tables S1 and S2.

## 4. Discussion

Diagnostic US is an indispensable modality in veterinary medicine, offering a noninvasive, real-time, and dynamic approach to assessing GI health and diagnosing intestinal diseases in dogs and cats (Gaschen 2011). Most GI pathologies, from inflammatory to neoplastic conditions, alter the thickness and integrity of intestinal wall layers. As a result, abdominal US has proven to be a valuable diagnostic tool for these frequently encountered diseases (Patsikas et al. 2003; Penninck et al. 2003; Stander et al., 2010; Gaschen 2011). Furthermore, histopathological diagnosis of intestinal inflammatory or neoplastic disease can be challenging when based on aspirate, endoscopic, or ultrasound-guided biopsy samples. Even with full-thickness surgical biopsies, there is still a risk of missing the affected area. This underscores the crucial role of accurate US diagnosis in such cases (Penninck et al. 2003). While assessing the overall thickness of the GI wall—from the inner mucosal interface to the outer serosa—one crucial aspect of abdominal ultrasound examinations is the detailed evaluation of individual wall layers (serosa, muscularis, submucosa, and mucosa), as this provides valuable clinically relevant information (Winter et al. 2014) (Figure 2). Thus, well-defined values and characteristics of the healthy GI tract are essential. These parameters allow veterinarians to identify deviations from normal more quickly and accurately.

Review of the literature on cats revealed detailed characterization of the GI tract (Winter et al. 2014) and cecum (Hahn et al. 2017). The absence of weight differentiation (as no significant differences were reported by weight (Martinez et al. 2018)) contributes to the statistical robustness of these studies, resulting in well-established reference values. These studies included cats aged 0.5 to 16 years (Winter et al. 2014), encompassing a broad spectrum from very young to elderly cats. This age range provides a broad representation of adult cats; however, the relatively small sample sizes still limit population-level generalization. Although gender information is often lacking and sex-based analyses are rare, this limitation has minimal impact because gender differences in body size and weight are small in cats. Regarding the stomach, variations in the thickness of the rugal folds compared to the inter-rugal regions highlight the importance of employing distinct evaluation standards. However, gastric distension does not markedly change the thickness of rugal folds or inter-rugal regions (Newell et al. 1999). Measurements taken throughout the intestine exhibit a relative uniformity across various studies, which underscores a level of consistency. This uniformity reinforces data reliability and supports their use as descriptive reference values to assist clinical interpretation. Moreover, it is worth highlighting the coherent approach employed in the acquisition of images. Whether captured longitudinally or transversely, there appears to be no significant differences between ultrasonographic measurements. This consistency in image acquisition strategies enhances the overall robustness of the data, facilitating a comprehensive evaluation of the feline GI tract.

Compared with feline studies, characterization of the GI tract in dogs remains less comprehensive. For example, sedation does not seem to significantly affect the measurement of the wall along the GI tract, however it did increase the full thickness of the duodenum (Newell et al. 1999). Another limitation is the absence of data from older animals and the lack of gender-based analysis, emphasizing the need for more diverse research that accounts for age-related and potential sex-related differences in canine GI morphology. None of the available studies classify animals by breed, although most differentiate them by weight (Penninck et al. 1989; Delaney et al. 2003; Stander et al., 2010; Gladwin et al. 2014). However, there is no consensus about the categories. Delaney et al. (Delaney et al. 2003) divided the dogs into five groups by weight while Gladwin et al. (Gladwin et al. 2014) divided into three groups. All these studies reported associations between GI wall thickness and body weight. Larger dogs tended to present thicker GI walls compared with smaller dogs. Nevertheless, as mentioned, this association was not uniformly evaluated across all studies, and differences in study design, population composition, and ultrasound protocols limit direct comparisons. Therefore, although body weight appears to be associated with wall thickness, a causal relationship cannot be inferred from the currently available evidence. Also, no study provides a complete characterization of the entire GI tract, and evaluation of the ileum is limited to a single report that did not consider body weight (Le Roux et al. 2016). Furthermore, it’s worth noting that in adult animals, the evaluation of the stomach was only conducted in one paper published in 1989 (Penninck et al. 1989). Because of the limited number of animals in each category, those authors advised that their data should be interpreted as preliminary reference points for future work (Penninck et al. 1989). Despite these gaps, descriptions of the intestinal wall and reference measurements for all GI segments—including total wall thickness and individual layers—are available (Tables 3 and 5).

Age-related associations were inconsistently reported among the included studies. While some authors observed slightly greater wall thickness in adult animals compared with juveniles, others did not detect significant differences. These discrepancies may reflect differences in sample size, population structure, or technical factors rather than true biological effects.

Another relevant methodological aspect concerns the variability in ultrasound probe frequency among the reviewed studies that ranged from 5 to 18 MHz. In feline studies (Newell et al. 1999; Goggin et al. 2000; Di Donato et al. 2014; Winter et al. 2014; Hahn et al. 2017; Martinez et al. 2018) higher-frequency probes (10–18 MHz) offered superior resolution, while canine studies (Penninck et al. 1989; Delaney et al. 2003; Stander et al., 2010; Gladwin et al. 2014; Le Roux et al. 2016; Banzato et al. 2017) often used lower frequencies (5–9 MHz) that allowed greater depth but less detail (Meomartino et al. 2021; Seiler et al. 2022). Such methodological differences probably contributed to interspecies discrepancies, particularly in gastric wall measurements. Also, scanning planes and measurement techniques were not standardized, and fasting protocols differed among studies. In addition, some studies were performed under sedation or anesthesia, while others were conducted in awake animals. These factors are known to influence GI motility and wall appearance and may therefore act as confounding variables.

The AXIS appraisal highlighted consistent methodological limitations across the evidence base that temper the certainty of the proposed reference intervals. In particular, the lack of inter-observer reproducibility data for ultrasound measurements and the frequent absence of sample-size justification reduce confidence in the precision and repeatability of some layer-specific values. *Ex-vivo* and cadaveric correlation studies strengthened criterion validity for layer identification but are limited in direct applicability to *in-vivo* imaging. Heterogeneity in equipment (transducer frequency), fasting/sedation protocols and population selection (referral samples, breed skew) probably explains part of the dispersion of reported values. Clinicians should therefore apply the proposed reference intervals as approximate guide-ranges and consider individual patient context and equipment settings when interpreting measurements

Differences in reported gastrointestinal wall thickness and echogenicity have direct biological and clinical implications. In practice, diagnostic interpretation depends on comparison with published reference ranges (Esteves-Monteiro et al. 2025); however, when these differ between studies, distinguishing normal from abnormal findings becomes uncertain. Even small variations—due to equipment settings, luminal distension, or animal size—can shift diagnostic thresholds and influence clinical judgment (Larson and Biller 2009; Stander et al., 2010). Overestimation of normal values may lead to underdiagnosis of early inflammatory or infiltrative processes, whereas underestimation may increase false-positive findings, prompting unnecessary further testing or treatment. In cats, for instance, intestinal wall thickness overlapping between healthy and mildly lymphoplasmacytic enteritis cases has been documented, highlighting the clinical challenge of relying on inconsistent reference data (Zwingenberger et al. 2010; Gaschen 2011). Similarly, in dogs, variations in reported gastric wall thickness have been attributed to differences in probe frequency and fasting state, affecting the detection of conditions such as gastritis or early neoplasia (Gaschen 2011; Seiler et al. 2022).

Recent studies further emphasize that evolving ultrasonographic technology and advanced imaging protocols continue to influence gastrointestinal measurements and diagnostic thresholds. For example, newer investigations into gastrointestinal wall changes in dogs with acute pancreatitis (Hardwick et al. 2022) and updated imaging reviews on neoplastic disease detection (Ercolin et al. 2024) both highlight how differences in wall thickness and layering patterns can influence diagnostic interpretation. Such findings may reflect underlying inflammatory or neoplastic processes. Although these patterns are not specific to a single condition, recognizing their variability within normal limits is essential for improving diagnostic confidence and reducing the risk of misinterpretation in clinical practice (Ercolin et al. 2024).

It is important to note that only twelve studies met the inclusion criteria for this review, and several of them were published more than a decade ago. This limited number of eligible studies reflects the scarcity of systematic research on GI US in small animals rather than a restriction of our search strategy. The synthesis of these available data remains highly relevant, as it provides a structured summary of the current evidence, identifies methodological inconsistencies, and emphasizes the need for updated and standardized studies to refine reference values and improve clinical applicability. Also, an extensive study that assesses the complete GI tract (stomach, duodenum, jejunum, ileum, and colon) with a substantial number of animals could be highly beneficial. Such a study could help standardize not only the weight categories but also the reference values for the overall thickness of the GI wall and its different layers. Research concerning puppies and the existence of reference values in this age group are particularly significant, given the prevalence of enteritis in this specific age range (Stander et al., 2010). In fact, it seems that there is an increase in the wall thickness of all the GI tracts during development (Banzato et al. 2017). However, there is no consensus between authors.

This systematic review has several important limitations that must be considered when interpreting the reported reference values. First, the limited number of eligible studies and the relatively small sample sizes in many reports reduce the precision and generalizability of the proposed reference ranges. Age was inconsistently controlled across studies, and the inclusion of both juvenile and adult animals may have influenced wall thickness measurements, potentially leading to inflation of reference values and reducing their applicability to specific age groups.

Body weight was another relevant confounding factor, particularly in dogs. Although most studies attempted to stratify animals by weight, the lack of standardized weight categories limits comparability between studies and may compromise the accurate application of reference intervals to intermediate-sized breeds. Similarly, breed was rarely reported or considered, despite known conformational differences that may influence GI anatomy.

Methodological heterogeneity represents a major source of bias. Marked variability in ultrasound equipment and transducer frequencies, scanning planes, fasting protocols, and measurement techniques limits direct interstudy comparisons. Lower-frequency probes, may underestimate the thickness of individual wall layers, particularly in large dogs, thereby reducing sensitivity for subtle pathological changes. In addition, the use of sedation or anesthesia in some studies but not in others may have influenced gastrointestinal motility, luminal distension, and apparent wall thickness, introducing further measurement bias.

In most studies, classification of animals as ‘clinically healthy’ was based on physical examination and routine laboratory testing rather than histopathological confirmation. Consequently, the presence of subclinical GI disease cannot be fully excluded, which may have shifted reported reference values toward higher measurements and reduced the contrast between healthy and pathological states. Also, definitions of ‘healthy’ and screening protocols varied substantially between studies, ranging from physical examination alone to comprehensive laboratory and histopathological assessment. This heterogeneity, summarized in Tables 1 and 2, limits direct comparability between studies and represents an inherent source of selection bias in the available literature.

Finally, selection and publication bias cannot be excluded. Many study populations were recruited from referral hospitals or academic settings and may not accurately represent the general companion animal population, particularly geriatric animals or those with multiple comorbidities. Together, these sources of bias indicate that the reference values compiled in this review should be interpreted as approximate descriptive intervals rather than absolute diagnostic thresholds. Future prospective studies using standardized imaging protocols, consistent population stratification, and integrated histopathological validation are essential to refine gastrointestinal ultrasonographic reference ranges and enhance their clinical applicability.

## 5. Conclusion

To accurately assess the GI tract of dogs and cats, this review systematically compiled current knowledge on its normal ultrasonographic appearance and established reference intervals for overall wall thickness and individual layers across GI segments in adult cats, dogs, and puppies. Qualitative ultrasonographic features of the GI tract are well described, but methodological variability still affects the consistency of quantitative findings. Establishing consensus-based reference ranges, as summarized in this review, represents a crucial step toward greater diagnostic reliability and more uniform interpretation of gastrointestinal ultrasonographic data.

In cats, the reviewed literature shows a high degree of coherence in both measurements and imaging techniques, providing a solid foundation for reference data that can serve as a baseline in clinical evaluation. In contrast, existing canine studies use inconsistent weight ranges, and there is no clear consensus regarding normal values across body sizes or sexes. Larger cohort studies that stratify dogs by weight and gender would help define consensual reference intervals and provide more precise diagnostic benchmarks. In addition, most research in dogs has focused on overall gastric wall thickness, leaving structures such as rugal folds and inter-rugal spaces underexplored. Because these features are integral to gastric function, targeted studies examining their ultrasonographic characteristics could enrich the understanding of normal anatomy and improve clinical interpretation.

Furthermore, while imaging techniques offer valuable measurements of GI thickness, only most of the cat studies have incorporated histopathological confirmation of these findings (Newell et al. 1999; Goggin et al. 2000; Hahn et al. 2017; Martinez et al. 2018), while in dogs only one study includes this component (Le Roux et al. 2016). Integrating imaging and microscopic analysis would enhance the reliability of GI assessment by linking wall thickness and layering patterns with true tissue architecture. Such correlation is essential for accurate diagnosis of inflammatory and neoplastic diseases.

Ultrasonographic evaluation of the GI tract in cats and dogs remains a cornerstone of veterinary practice, providing a noninvasive, repeatable, and highly informative method for assessing structural and functional abnormalities. This review highlights that while ultrasonography provides reliable structural assessment of the gastrointestinal tract, its diagnostic accuracy remains highly dependent on methodological standardization and population-specific reference values.

## Supplementary Material

Supplemental Material
