# Supplementary material for: Ultrasonographic evaluation of the normal gastrointestinal wall in dogs and cats: a systematic review on study design and imaging outcomes
Source: Vet Q. 2026 Feb 9;46(1):2622732. doi: 10.1080/01652176.2026.2622732 (PMC12888347; doi:10.1080/01652176.2026.2622732)
Supplement: Supplemental Material [file TVEQ_A_2622732_SM8017.docx]

**Supplementary files**

**Supplementary Table S1.** AXIS appraisal (20 items) for each included study in cats. For each study each AXIS item is scored as Yes / Partial / No / Unclear; one-line justifications for each score are provided. “Partial” means the AXIS question was addressed but incompletely (e.g., eligibility criteria described but some items missing). “Unclear” means the manuscript provides insufficient information to judge.

|  | **Newell et al., 1999** | **Goggin et al., 2000** | **Winter et al., 2013** | **Di Donato et al., 2014** | **Hahn et al., 2017** | **Martinez et al., 2018** |
| --- | --- | --- | --- | --- | --- | --- |
| **Introduction** | | | | | | |
| Clear aims? | Yes | Yes | Yes | Yes | Yes | Yes |
| **Methods** | | | | | | |
| Appropriate design? | Yes, cross-sectional descriptive study appropriate. | Yes | Yes | Yes | Yes | Yes |
| Sample size justification? | No, no sample size justification; small N (n=14). | No, no sample size justification; small initial N (n≈11, 9 after histology). | Partial: N=38 reasonable for descriptive study but no formal power calculation. | No: no formal sample-size justification; sample n=20. | No: small sample (n=20) and no sample-size calculation. | No: no sample-size justification (n=17 cadavers). |
| Target population defined? | Partial: population described (age range) but gender/weights missing. | Partial: population criteria described but age/gender incompletely reported. | Yes: inclusion criteria (clinically healthy, lab tests) clearly reported. | Yes: inclusion/exclusion criteria and health screening reported (CBC, biochemistry, fecal). | Yes: inclusion criteria specified (no GI signs, clinical screening). | Partial: cadaver population described, but full *ante-mortem* histories limited. |
| Selection methods described? | Partial: selection described (healthy), but incomplete criteria | Partial: recruitment method unclear | Yes: inclusion criteria (clinically healthy, lab tests) clearly reported | Yes: measurement technique, probe, planes, fasting and manual restraint detailed | Yes: methods for US, endoscopy, histology described; sedation/GA specified. | Yes: clear measurement methods for US and histology; timing to fixation noted. |
| Non-responders described? | No: non-responders / excluded not discussed. | No: non-responders/excluded not discussed. | No: non-responders/excluded not discussed. | No: non-responders/excluded not discussed. | No: non-responders/excluded not discussed. | No: non-responders/excluded not discussed. |
| Measures and methods clearly defined? | Yes | Yes | Yes | Yes | Yes | Yes |
| Valid/reliable measurements? | Partial: probe frequency and planes stated but interobserver not reported. | Partial: probe frequency reported; anesthesia effects acknowledged but not quantified. | Partial: probe frequencies given; authors note spatial resolution limits for thin layers. | Partial: measurements appropriate for probe used; interobserver variability not quantified. | Yes — histologic correlation increases measurement validity; kappa used for agreement. | Yes: strong histologic validation for many layers; note of *post-mortem* changes affecting measurements. |
| Statistical methods described? | Partial: simple stats reported; methods limited. | Partial: descriptive stats used; no sample-size/power analysis. | Yes | Yes | Partial: appropriate agreement stats (κ value) used; full statistical breadth limited by sample size | Yes |
| Sample representative? | Partial: limited representativeness. | Partial: small, likely referral sample; representativeness limited. | Partial: sample reasonably varied but convenience sampling limits population representativeness. | Partial: single-centre convenience sample; representativeness limited. | No: sample skewed (majority Siamese intact males – 10/20); not representative. | Partial: cadaver sample limits representativeness for live animals. |
| Confounding identified & accounted? | Partial: some confounders discussed (distension, sedation) but not fully controlled. | Partial: authors note anesthesia as potential confounder but do not adjust analytically. | Partial: some confounders (diet) explored; others not fully adjusted. | Partial: operator influence acknowledged; limited confounder adjustment. | Partial: potential confounders discussed (breed skew) but limited analytical control. | Partial — *post-mortem* interval and fixation discussed as confounders; no analytical control possible. |
| **Results** | | | | | | |
| Main findings clearly described? | Yes | Yes | Yes | Yes | Yes | Yes |
| Precision estimates reported? | Partial: some CIs given for select measures; not universal. | Partial: ranges provided; CIs not routinely reported. | Yes | Partial: ranges and means given; formal CIs limited. | Partial: κ value presented; broader precision estimates limited by N. | Yes: correlation coefficients given; formal CIs for all measures variables. |
| Internal consistency / interobserver reported? | No: no interobserver / intraobserver metrics | No: no inter/intra-observer reproducibility reported. | No: inter- and intra-observer reliability not reported. | No: inter/intra-observer reproducibility not reported. | Partial: agreement metrics provided (κ value) but sample small to robustly assess reproducibility. | Partial: agreement between US and histology reported; interobserver on US not fully detailed. |
| Results consistent with aims? | Yes: results align to aims. | Yes | Yes | Yes | Yes | Yes |
| **Discussion** | | | | | | |
| Discussion of limitations? | Yes: authors discuss small N and other limitations. | Yes: limitations (low N, positioning effects) discussed. | Yes: authors discuss limitations (resolution limits, diet variability. | Yes: limitations (observer dependence, small sample) discussed. | Yes: limitations (small N, breed bias) explicitly discussed. | Yes: limitations related to *post-mortem* interval and fixation acknowledged. |
| Generalizability discussed? | Partial: generalizability to wider cat populations is cautioned. | Partial: generalizability limited by small sample and methods. | Partial: generalizability to all cat populations is cautious given sampling and equipment variability. | Partial: generalizability limited to similar clinical settings. | No: generalizability limited due to breed/sex skew and small sample. | Partial: generalizability to *in-vivo* settings explicitly limited. |
| Conclusions supported by data? | Yes: conclusions appropriately limited by data. | Yes: conclusions appropriate given presented data. | Yes | Yes | Yes | Yes |
| **Other** | | | | | | |
| Ethics approval / consent reported? | Yes | Yes | Yes | Yes | Yes | Yes |
| Funding/competing interests disclosed? | Unclear: funding/COI not clearly stated in main text. | Unclear — funding/COI not clearly stated. | Yes | Yes | Yes | Unclear — funding/COI not clearly stated. |

CI: confidence interval.

**Supplementary Table S2.** AXIS appraisal (20 items) for each included study in dogs. For each study each AXIS item is scored as Yes / Partial / No / Unclear; one-line justifications for each score are provided. “Partial” means the AXIS question was addressed but incompletely (e.g., eligibility criteria described but some items missing). “Unclear” means the manuscript provides insufficient information to judge.

|  | **Penninck et al., 1989** | **Delaney et al., 2003** | **Stander et al., 2010** | **Gladwin et al., 2014** | **Le Roux et al., 2016** | **Banzato et al., 2017** |
| --- | --- | --- | --- | --- | --- | --- |
| **Introduction** | | | | | | |
| Clear aims? | Yes | Yes | Yes | Yes | Yes | Yes |
| **Methods** | | | | | | |
| Appropriate design? | Yes | Yes | Yes | Yes | Yes | Yes |
| Sample size justification? | No: small numbers per group; no sample-size statement (older paper). | Partial: large sample (n=231) confers precision, but no formal power calculation reported. | No: small sample (n=23) and no power justification. | Partial: sample size moderate (n=85) but no formal power calculation. | No: small sample (n=12) and no sample-size calculation. | Partial: overall sample moderate (n=84 across timepoints) but per-cell N smaller and no formal power calculation. |
| Target population defined? | Partial: groups defined by weight/age but small N per category. | Yes: target population described and grouped by weight. | Yes: age and weight ranges specified. | Yes: adult dogs defined; weight groups described. | Partial: specimens described (weights/age) but *ex-vivo* nature limits target-population clarity. | Yes: age groups and weights defined (4, 8, 16 weeks). |
| Selection methods described? | Partial: methods (planes, probes, distension assessment) described but detail limited by standards of era. | Partial: selection by clinical exam/history; recruitment approach (convenience) implied. | Yes: methods (planes, probes, fasting) described but compliance affected data completeness. | Yes: measurement protocol described (single transverse image, layer measures). | Yes: methods for US *ex-vivo* and histology described. | Yes: methods (planes, probe, fasting) described; repeated measures across ages. |
| Non-responders described? | No: non-responders/exclusions not discussed. | No: non-responders/exclusions not discussed. | No: non-responders/missing data not systematically characterized. | No: non-responders/exclusions not described. | No: non-responders/exclusions not described. | No: missing measures due to noncompliance not systematically reported as non-responders. |
| Measures and methods clearly defined? | Yes | Yes | Yes | Yes | Yes | Yes |
| Valid/reliable measurements? | Partial: probe frequencies appropriate for era; modern resolution limitations apply. | Partial: probe ranges given; some segments (duodenum) not identified in all dogs, affecting validity for those cases. | Partial: probe frequencies given; measurement quality affected by aerophagia and movement. | Partial: probe details present; measurement by single observer raises reliability concerns. | Partial: good correlation for many layers; serosa correlation weaker and resolution limits acknowledged. | Partial: probe range adequate; measurement completeness affected by compliance and aerophagia. |
| Statistical methods described? | Partial: descriptive statistics but limited formal precision metrics. | Yes: statistical methods to compare groups described. | Partial: descriptive stats used; missing data issues noted. | Yes: descriptive statistics and group comparisons appropriate. | Yes: correlation statistics and agreement measures reported. | Yes: descriptive stats and age comparisons reported. |
| Sample representative? | Partial: small, experimental groups limit representativeness. | Yes: large and wide weight range improves representativeness despite convenience sampling. | Partial: limited representativeness due to small N and compliance issues. | Yes | Partial: ex-vivo sample not representative of in-vivo clinical population. | Partial: developmental coverage good; representativeness limited by missing data and clinic sampling. |
| Confounding identified & accounted? | Partial: distension and relaxation considered; confounding not analytically controlled. | Partial: potential confounders (duodenal identification) acknowledged; formal multivariable adjustment limited. | Partial: aerophagia and movement acknowledged as confounders but not analytically addressed. | Partial: potential confounders (breed, operator) not fully adjusted. | Partial: fixation, devitalization effects discussed as confounders; not analytically adjustable. | Partial: confounders (movement, aerophagia) acknowledged; not analytically corrected. |
| **Results** | | | | | | |
| Main findings clearly described? | Yes | Yes | Yes | Yes | Yes | Yes |
| Precision estimates reported? | Partial: ranges given; formal CIs generally absent (paper predates common CI reporting). | Yes: means and ranges reported | Partial: means/ranges provided; CI reporting limited. | Yes: ranges and means provided | Yes: correlation coefficients reported; some layer discrepancies noted and quantified. | Yes |
| Internal consistency / interobserver reported? | No: reproducibility metrics not reported. | No: interobserver reliability not described. | No: reproducibility metrics not reported. | No: no interobserver reliability; single measurer. | Partial: measurement repeats and correlation reported; explicit interobserver metrics limited. | No: interobserver reproducibility not reported. |
| Results consistent with aims? | Yes | Yes | Yes | Yes | Yes | Yes |
| **Discussion** | | | | | | |
| Discussion of limitations? | Yes: authors note limitations (small samples, gas interference). | Yes | Yes: limitations (compliance, missing measures) discussed. | Yes: limitations (single measurer, small per-group N) discussed. | Yes: limitations (ex-vivo vs in-vivo differences) clearly discussed. | Yes: limitations (compliance, missing measures) clearly discussed. |
| Generalizability discussed? | Partial: generalizability limited by era, small groups. | Partial: generalizability good for weight strata but breed/sex details limited. | Partial: generalizability limited to compliant pediatric patients. | Partial: generalizability reasonable but limited by single-operator uncertainty. | No: generalizability to live dogs explicitly limited. | Partial: generalizability to broader puppy populations limited by missing data. |
| Conclusions supported by data? | Yes | Yes | Yes | Yes | Yes | Yes |
| **Other** | | | | | | |
| Ethics approval / consent reported? | Unclear: ethics statements less common in 1989; not explicit. | Unclear: clinical screening performed; ethics statement not explicit. | Unclear: basic clinical screening described; explicit ethics statement unclear in excerpt. | Unclear: clinical screening performed; ethics statement not explicit. | Unclear: specimens handled appropriately; explicit ethics wording limited in excerpt. | Unclear: clinical screening described; explicit ethics statement not prominent in excerpt. |
| Funding/competing interests disclosed? | No | No | No | No | No | Yes |

CI: confidence interval.
